# Can we talk about microglia without neurons? A discussion of microglial cell autonomous properties in culture

**DOI:** 10.3389/fncel.2014.00202

**Published:** 2014-07-24

**Authors:** Ismael Neiva, João O. Malva, Jorge Valero

**Affiliations:** ^1^Neuroprotection and Neurogenesis in Brain Repair Group, Center for Neuroscience and Cell Biology, University of CoimbraCoimbra, Portugal; ^2^Faculty of Medicine, Institute of Biomedical Imaging and Life Sciences, University of CoimbraCoimbra, Portugal; ^3^Institute for Interdisciplinary Research, University of CoimbraCoimbra, Portugal

**Keywords:** cell culture, CX3CR1, fractalkine, microglia, N9 cells

## Microglial origin and function

Microglial cells originate from precursor cells located in the yolk sac that migrate into the developing central nervous system (CNS) around E8.5-10 in mice. Considering their origin, microglial cells could be regarded as invaders of the CNS in charge of debris clearance, just active during brain injury, infection or degeneration. In adulthood and under certain conditions, monocytes may penetrate the blood brain barrier, reach the CNS and become non-resident brain macrophages. CNS infiltrating-macrophages are difficult to distinguish from resident microglial cells and it is not clear the grade of functional similarity between these two types of cells (Prinz and Mildner, 2011; Gomez Perdiguero et al., 2013). Interestingly, microglial cells are able to self-renew independently of circulating monocytes or bone marrow hematopoietic stem cells (Ajami et al., 2007; Elmore et al., 2014), and show a specific molecular signature (Butovsky et al., 2014). Moreover, during the last decade microglial cells have been demonstrated to be involved in normal brain development and function while maintaining a, previously assumed, “resting” state (Pont-Lezica et al., 2011; Tremblay et al., 2011; Valero et al., 2012). Thus, microglial cells cannot be considered just as macrophages.

## The actively “resting” microglia and their interaction with neurons

The healthy brain is continuously under the active surveillance of a dynamic network of microglial processes. These processes have been shown to permanently protrude and retract, and to physically interact with neuronal synapses to modulate synaptic plasticity. Microglia phagocytic control of synaptic pruning is mediated by the complement pathway (Schafer et al., 2012) and fractalkine signaling (Paolicelli et al., 2011). Furthermore, microglia also regulate synaptic plasticity by the release of diffusible molecules like the brain-derived neurotrophic factor (Parkhurst et al., 2013) or the coactivator of synaptic NMDA receptors D-serine (Scianni et al., 2013). Therefore, microglial cells are able to modulate neuronal function in several ways. Importantly, this is not a unidirectional path of communication, but a dialog between microglia and neurons (Saijo and Glass, 2011; Eyo and Wu, 2013). As a clear example, the chemokine fractalkine (constitutively expressed by neurons), and its receptor (CX3CR1, mainly present in microglial cells), participate in the modulation of hippocampal synaptic pruning and function (Paolicelli et al., 2011; Scianni et al., 2013; Zhan et al., 2014). Fractalkine/CX3CR1 axis is better known to be involved in keeping microglia in their “resting” surveillance state (Sheridan and Murphy, 2013; Wolf et al., 2013). Thus, fractlakine/CX3CR1 system represents a clear example of mutual functional regulation between neurons and microglia. Moreover, there are other pairs of neuronal ligands and microglial receptors which mediate communication between these two elements of the CNS: the neuronal surface proteins CD200, CD47, and CD22 that bind to the microglial receptors CD200R, CD172, and CD45, respectively (Biber et al., 2007).

Microglial cells show regional differences in the brain in terms of density, molecular characteristics, morphology, and responsiveness (Olah et al., 2011; Butovsky et al., 2014). All microglial cells express the colony-stimulating factor receptor (CSF1R), which is required for their development. Interleukine-34, a ligand of CSF1R, is mainly expressed by neurons in specific regions of the brain (cortex, hippocampus, striatum, and anterior olfactory nucleus) where it promotes microglial survival (Greter et al., 2012; Wang et al., 2012). Similarly, high levels of fractalkine expression have been found in neurons of the amygdala, striatum, globus pallidus, thalamus, olfactory bulb, hippocampus, and cerebral cortex (Tarozzo et al., 2003; Kim et al., 2011). Fractalkine is also expressed by astrocytes at lower levels (Hulshof et al., 2003; Sunnemark et al., 2005). Interestingly, the specific molecular signature of adult microglia is dependent on the presence of the transforming growth factor-β (TGF-β) which is expressed at low levels by neurons and glial cells (Butovsky et al., 2014). Therefore, neurons, by producing different levels of the aforementioned molecules, may be responsible of the regional characteristics and heterogeneity of microglial cells.

## Microglial cells in the absence of neurons

Taking into account previous data, it seems clear that neurons are active modulators of microglial functional state and could be responsible for microglial regional differences. Considering that, under normal conditions, neurons shape microglial cells and that the main role of microglia is the functional modulation and maintenance of neurons, an important question emerges: does it make sense to talk about microglia in the absence of neurons? Indeed, this question can be re-formulated as: do microglial cells retain their identity in the absence of neurons? If we consider that the interaction with neurons is crucial for the definition of microglia, the answer seems to be clear. Our opinion paper aims to provoke reflection and is in no way intended to despise the value of prior studies that, using isolated microglial cells or cell lines, have contributed to the current knowledge of microglial cell biology.

**Figure 1:**
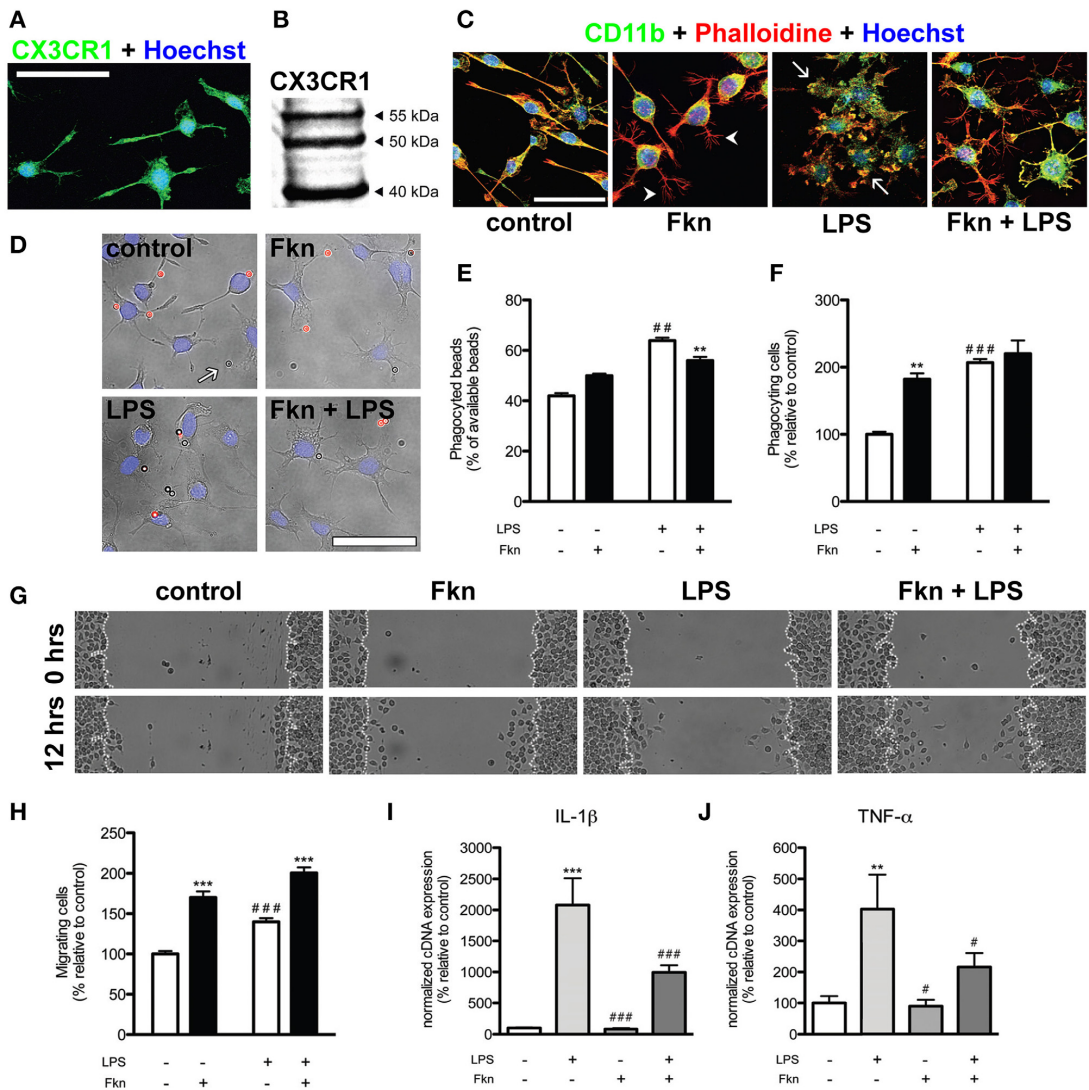
**Soluble fractalkine changes morphology and behavior of N9 microglial cells. (A)** Confocal microscopy image of N9 microglial cells expressing CX3CR1 (green, rabbit anti-CX3CR1, eBioscience). Cell nuclei were stained with Hoechst 33342 (blue, Life Technologies). Almost all N9 cells in basal culture conditions showed expression of CX3CR1. **(B)** Western blotting of 50 μ g whole cell extracts from N9 cells showing the previously described pattern (Yang et al., 2007). **(C)** Representative confocal images of N9 microglial cells stained for CD11b (green, rat anti-CD11b, AbD Serotec), filamentous actin (red, phalloidin-Alexa Fluor® 594 conjugate, Life Technologies) and Hoechst 33342 (blue). In basal (control) conditions N9 microglial cells showed different morphologies (from bipolar to ramified). Treatment of N9 cells with fractalkine chemokine domain (Fkn, 6 h, 200 ng/ml, Sigma-Aldrich) induced the adoption of a ramified morphology by N9 cells characterized by thin filopodia-like structures (arrowheads). As expected, lipopolysaccharide treatment (LPS, 6 h, 100 ng/ml, from *Escherichia coli*, Sigma-Aldrich) induced swelling, loss of ramified morphology and the appearance of thick membrane protrusions (“ruffles,” arrows) in N9 microglia. In the presence of both LPS and Fkn, N9 cells displayed an intermediate morphology showing “ruffles” and filopodia-like structures. **(D)** Representative epifluorescence/phase contrast images of N9 cells (cell nuclei stained with Hoechst 33342, blue) in the presence of beads (2 × 10^6^ beads/well, Sigma-Aldrich) coated with IgG from rabbit serum (Sigma-Aldrich). Due to the protocol used (for details check: Ferreira et al., 2011) phagocyted beads were not stained (arrows) while non-phagocyted beads showed red fluorescence (Alexa Fluor® 594 donkey anti-rabbit IgG, Life Technologies). **(E)** LPS treatment (6 h) led to an increase in the total number of phagocyted beads. Incubation with Fkn (6 h) just slightly reduced the percentage of beads phagocyted by LPS treated N9 cells. **(F)** LPS and Fkn treatments (6 h) increased the proportion of phagocyting cells. **(G,H)** A scratch wound assay was carried out to analyse N9 migratory activity (for details see: Ferreira et al., 2012) based on the mean number of cells that moved into the wound after 12 h. **(G)** Phase-contrast representative images of the scratch wound assay. **(H)** Co-incubation of N9 cells with LPS and Fkn resulted in a greater migratory induction than that elicited by LPS treatment alone. **(I)** IL-1β and **(J)** TNF-α mRNA expression in N9 cells was increased in the presence of LPS (4 h). Importantly Fkn treatment downregulated the increase in IL-1β and TNF-α mRNAs induced by LPS. All scale bars = 50 μm. Data are presented as mean ± s.e.m. **(E,F,H)**: ^**^p < 0.01 and ^***^p < 0.001 vs. respective condition without Fkn, ^##^*p* < 0.01 and ^###^*p* < 0.001 vs. control condition (2-Way ANOVA and Bonferroni's *post-hoc* test). **(I,J)**: ^**^p < 0.01 and ^***^p < 0.001 vs. control condition, ^#^*p* < 0.05 and ^###^*p* < 0.001 vs. LPS (Pair Wise Fixed Reallocation Randomization Test©, Pfaffl et al., 2002).

It is important to note that isolated microglia do not display, in cell cultures, the highly ramified structure that is typically observed in the normal, healthy CNS. *Ex vivo* microglial cells analyzed immediately upon isolation resemble reactive amoeboid microglia, probably due to the isolation process itself. Nevertheless, this activation seems to wane with time and cell passages, reaching an approximation to the typical “resting” morphology. Immortalized microglial cell lines are an alternative to the use of primary microglial cell cultures, which are costly and time consuming. Several microglial cell lines derived from rat (HAPI), mouse (BV2, N9, EOC), and also human (HMO6), have been used. These cell lines exhibit characteristic microglial/macrophage cell markers and behaviors (cytokine release, migration, and phagocytosis) in the presence of endotoxins such as the gram-negative bacteria lipopolysaccharide (LPS, Figure 1). However, the immortalization renders these cells different from primary microglia; in terms of molecular expression (Butovsky et al., 2014), morphology, proliferation, and adhesion (reviewed in Stansley et al., 2012).

## Filling the gap between *in vitro* and *in vivo* microglia

It still remains to be elucidated whether isolated microglial cells of *in vitro* systems behave as proper microglia. The importance of this question probably depends on the parameters to be evaluated, e.g., in the absence of direct contact with neurons these cells will fail to develop some specific characteristics of microglial cells. The obvious solution for the aforementioned problem is the use of co-culture systems in which microglial cells and neurons co-exist. Again, the regional origin of microglial and neuronal cells may influence their interaction and should be taking into account when inferring general mechanisms. Nevertheless, some studies require the use of isolated microglia. As previously mentioned, neurons release several factors that regulate microglial state. The use of the adequate combination of these factors in cell culture could contribute to the reduction of the gap between *in vivo* and *in vitro* systems, even in the absence of neurons. On this respect, we have investigated the effects of treating N9 microglial cells with soluble fractalkine in basal conditions or after activation with LPS. We observed that N9 cells expressed CX3CR1 mRNA and protein using quantitative real time PCR (not shown), immunofluorescence (Figure 1A) and western blotting (Figure 1B). Interestingly, in basal and LPS conditions fractalkine induced N9 cells to acquire a more ramified morphology (Figure 1C). Fractalkine treatment also increased the number of phagocyting (Figures 1D–F) and migrating N9 cells (Figures 1G,H). Furthermore, fractalkine was also able to reduce the expression of interleukin-1β (IL-1β) and tumor necrosis factor-α (TNF-α) mRNAs induced by LPS treatment (Figures 1I,J) while maintaining elevated levels of phagocytic and migratory activity in N9 cells. Fractalkine has a clear role in shaping microglial cells, evident at the morphological, functional and molecular levels. This role could be carried out by the membrane bound or even the constitutively cleaved fractalkine *in vivo*, keeping the cells in an alerted but relatively latent state (Sheridan and Murphy, 2013; Wolf et al., 2013). Thus, the lack of fractalkine in isolated microglial cultures is a possible reason for the lack of “surveying”-like microglial phenotype observed in these systems. Our data suggest that supplementation of microglial culture media with fractalkine may serve to shorten the gap between the typical morphology and behavior of microglia that is observed *in vivo* versus displayed in most *in vitro* models. Nevertheless, the addition of fractalkine to cell cultures is far from being a definitive solution. Therefore, this idea could be extended to the use of the adequate combination of microglial modulating factors released by neurons and/or macroglial cells. Butovsky et al. (2014) observed that adult microglia cultured in the presence of MCSF (macrophage colony-stimulating factor) and TGF-β 1 showed a molecular expression pattern similar to freshly sorted adult microglia. Nevertheless, treatment of N9 and BV2 cells with these factors did not induce the expression of such microglial molecular pattern (Butovsky et al., 2014), indicating the limitations of microglial cell lines. Thus, further research should be done to identify and define the individual contribution and combined effect of different factors to maintain the functional and molecular characteristics of microglial cells.

We must assume that the perfect *in vitro* system is just a utopia. By definition, *in vitro* microglia systems will never reach the complexity of *in vivo* ones, but in turn will allow the study of some aspects of microglial biology that are masked by surrounding factors (e.g. by the presence of neurons and/or macroglial cells). Thus, some intrinsic characteristics of microglia can be easier analyzed *in vitro*, like the molecular mechanisms involved in phagocytosis, cell migration, transcriptional control, and metabolic functioning. In the other way around, the analysis of complex systems can mask particular aspects of its individual components (microglial cells), but lead to the discovery of new mechanisms and functionalities that emerge from the interaction of the parts (e.g., microglial regulation of synaptogenesis). However, even *in vivo* experimental designs, due to the need of controlling as many variables as possible and to the use of experimental manipulations, are normally far from the complexity of natural systems. Thus, we should be cautious when trying to predict, from our particular experimental systems, the features and behavior of microglial cells in their natural milieu (a CNS integrated into a full alive organism, which is also influenced by its surrounding environment).

## Conclusions

Our initial question could be seen just as a mere language issue related to how we define “microglia”: (1) based on their intrinsic properties or (2) based on the specific characteristics that emerge through their interaction with other elements of the CNS. We consider that microglia are defined by these two aspects of their biology and that one influences the other. As an example, the constitutive expression of CX3CR1 by microglial cells, an intrinsic property shared with other macrophages, allows them to be shaped by neuronal fractalkine. As previously mentioned, the binding of fractalkine to its receptor controls microglial surveillance state but also mediates their role on the modulation of synaptic function, a specific characteristic that emerges through their interaction with neurons. Therefore, the study of intrinsic features (maybe shared with other cell types) and specific characteristics of microglia that emerge through their interaction with other components of the CNS are equally important to understand the nature of these cells. Obviously, these two ways of studying microglia will benefit in different grades from distinct experimental approaches, ranging from the examination of isolated cells *in vitro* to their analysis in their natural environment *in vivo*. Again, we will just need to be careful when generalizing or directly translating our observations from one to another level of biological complexity. These considerations will be specially relevant for the development of strategies aimed to use microglial cells as cell therapeutic agents.

## Author contributions

Conceived and designed the experiments: Ismael Neiva, João O. Malva, and Jorge Valero. Performed the experiments: Ismael Neiva, Analyzed the data: Ismael Neiva. Prepared figures: Ismael Neiva and Jorge Valero. Wrote the paper: Ismael Neiva, João O. Malva, and Jorge Valero.

### Conflict of interest statement

The authors declare that the research was conducted in the absence of any commercial or financial relationships that could be construed as a potential conflict of interest.

## References

[B1] Ajami B. Bennett J. L. Krieger C. Tetzlaff W. Rossi F. M. V. (2007). Local self-renewal can sustain CNS microglia maintenance and function throughout adult life. Nat. Neurosci. 10, 1538–1543 10.1038/nn2014 18026097

[B2] Biber K. Neumann H. Inoue K. Boddeke H. W. G. M. (2007). Neuronal “On” and “Off” signals control microglia. Trends Neurosci. 30, 596–602 10.1016/j.tins.2007.08.007 17950926

[B3] Butovsky O. Jedrychowski M. P. Moore C. S. Cialic R. Lanser A. J. Gabriely G. (2014). Identification of a unique TGF-β-dependent molecular and functional signature in microglia. Nat. Neurosci. 17, 131–143 10.1038/nn.3599 24316888 PMC4066672

[B4] Elmore M. R. P. Najafi A. R. Koike M. A. Dagher N. N. Spangenberg E. E. Rice R. A. (2014). Colony-stimulating factor 1 receptor signaling is necessary for microglia viability, unmasking a microglia progenitor cell in the adult brain. Neuron 82, 380–397 10.1016/j.neuron.2014.02.040 24742461 PMC4161285

[B5] Eyo U. B. Wu L.-J. (2013). Bidirectional microglia-neuron communication in the healthy brain. Neural Plast. 2013 10.1155/2013/456857 24078884 PMC3775394

[B6] Ferreira R. Santos T. Cortes L. Cochaud S. Agasse F. Silva A. P. (2012). Neuropeptide Y inhibits interleukin-1 beta-induced microglia motility. J. Neurochem. 120, 93–105 10.1111/j.1471-4159.2011.07541.x 22007767

[B7] Ferreira R. Santos T. Viegas M. Cortes L. Bernardino L. Vieira O. V. (2011). Neuropeptide Y inhibits interleukin-1β-induced phagocytosis by microglial cells. J. Neuroinflammation 8:169 10.1186/1742-2094-8-169 22136135 PMC3239417

[B8] Gomez Perdiguero E. Schulz C. Geissmann F. (2013). Development and homeostasis of “resident” myeloid cells: the case of the microglia. Glia 61, 112–120 10.1002/glia.22393 22847963

[B9] Greter M. Lelios I. Pelczar P. Hoeffel G. Price J. Leboeuf M. (2012). Stroma-derived interleukin-34 controls the development and maintenance of langerhans cells and the maintenance of microglia. Immunity 37, 1050–1060 10.1016/j.immuni.2012.11.001 23177320 PMC4291117

[B10] Hulshof S. van Haastert E. S. Kuipers H. F. van den Elsen P. J. De Groot C. J. van der Valk P. (2003). CX3CL1 and CX3CR1 expression in human brain tissue: noninflammatory control versus multiple sclerosis. J. Neuropathol. Exp. Neurol. 62, 899–907 14533779 10.1093/jnen/62.9.899

[B11] Kim K.-W. Vallon-Eberhard A. Zigmond E. Farache J. Shezen E. Shakhar G. (2011). *In vivo* structure/function and expression analysis of the CX3C chemokine fractalkine. Blood 118, e156–e167 10.1182/blood-2011-04-348946 21951685 PMC4507037

[B12] Olah M. Biber K. Vinet J. Boddeke H. W. (2011). Microglia phenotype diversity. CNS Neurol. Disord. Drug Targets 10, 108–118 10.2174/187152711794488575 21143141

[B13] Paolicelli R. C. Bolasco G. Pagani F. Maggi L. Scianni M. Panzanelli P. (2011). Synaptic pruning by microglia is necessary for normal brain development. Science 333, 1456–1458 10.1126/science.1202529 21778362

[B14] Parkhurst C. N. Yang G. Ninan I. Savas J. N. Yates J. R. Lafaille J. J. (2013). Microglia promote learning-dependent synapse formation through BDNF. Cell 155, 1596–1609 10.1016/j.cell.2013.11.030 24360280 PMC4033691

[B15] Pfaffl M. W. Horgan G. W. Dempfle L. (2002). Relative expression software tool (REST©) for group-wise comparison and statistical analysis of relative expression results in real-time PCR. Nucleic Acids Res. 30, e36–e36 10.1093/nar/30.9.e36 11972351 PMC113859

[B16] Pont-Lezica L. Béchade C. Belarif-Cantaut Y. Pascual O. Bessis A. (2011). Physiological roles of microglia during development. J. Neurochem. 119, 901–908 10.1111/j.1471-4159.2011.07504.x 21951310

[B17] Prinz M. Mildner A. (2011). Microglia in the CNS: immigrants from another world. Glia 59, 177–187 10.1002/glia.21104 21125659

[B18] Saijo K. Glass C. K. (2011). Microglial cell origin and phenotypes in health and disease. Nat. Rev. Immunol. 11, 775–787 10.1038/nri3086 22025055

[B19] Schafer D. P. Lehrman E. K. Kautzman A. G. Koyama R. Mardinly A. R. Yamasaki R. (2012). Microglia sculpt postnatal neural circuits in an activity and complement-dependent manner. Neuron 74, 691–705 10.1016/j.neuron.2012.03.026 22632727 PMC3528177

[B20] Scianni M. Antonilli L. Chece G. Cristalli G. Castro M. A. D. Limatola C. (2013). Fractalkine (CX3CL1) enhances hippocampal N-methyl-d-aspartate receptor (NMDAR) function via d-serine and adenosine receptor type A2 (A2AR) activity. J. Neuroinflammation 10:108 10.1186/1742-2094-10-108 23981568 PMC3765929

[B21] Sheridan G. K. Murphy K. J. (2013). Neuron–glia crosstalk in health and disease: fractalkine and CX3CR1 take centre stage. Open Biol. 3:130181 10.1098/rsob.130181 24352739 PMC3877844

[B22] Stansley B. Post J. Hensley K. (2012). A comparative review of cell culture systems for the study of microglial biology in Alzheimer's disease. J. Neuroinflammation 9:115 10.1186/1742-2094-9-115 22651808 PMC3407712

[B23] Sunnemark D. Eltayeb S. Nilsson M. Wallström E. Lassmann H. Olsson T. (2005). CX3CL1 (fractalkine) and CX3CR1 expression in myelin oligodendrocyte glycoprotein-induced experimental autoimmune encephalomyelitis: kinetics and cellular origin. J. Neuroinflammation 2:17 10.1186/1742-2094-2-17 16053521 PMC1188067

[B24] Tarozzo G. Bortolazzi S. Crochemore C. Chen S.-C. Lira A. S. Abrams J. (2003). Fractalkine protein localization and gene expression in mouse brain. J. Neurosci. Res. 73, 81–88 10.1002/jnr.10645 12815711

[B25] Tremblay M.-È. Stevens B. Sierra A. Wake H. Bessis A. Nimmerjahn A. (2011). The role of microglia in the healthy brain. J. Neurosci. 31, 16064–16069 10.1523/JNEUROSCI.4158-11.2011 22072657 PMC6633221

[B26] Valero J. Eiriz M. F. Santos T. Neiva I. Ferreira R. Malva J. O. (2012). Microglia: the bodyguard and the hunter of the adult neurogenic niche, in Advances in Stem Cell Research Stem Cell Biology and Regenerative Medicine, eds Baharvand H. Aghdami N. (Humana Press), 245–279 Available online at: http://link.springer.com/chapter/10.1007/978-1-61779-940-2_14 (Accessed May 12, 2013).

[B27] Wang Y. Szretter K. J. Vermi W. Gilfillan S. Rossini C. Cella M. (2012). IL-34 is a tissue-restricted ligand of CSF1R required for the development of Langerhans cells and microglia. Nat. Immunol. 13, 753–760 10.1038/ni.2360 22729249 PMC3941469

[B28] Wolf Y. Yona S. Kim K.-W. Jung S. (2013). Microglia, seen from the CX3CR1 angle. Front. Cell. Neurosci. 7:26 10.3389/fncel.2013.00026 23507975 PMC3600435

[B29] Yang X. P. Mattagajasingh S. Su S. Chen G. Cai Z. Fox-Talbot K. (2007). Fractalkine upregulates intercellular adhesion molecule-1 in endothelial cells through CX3CR1 and the JAK–STAT5 pathway. Circ. Res. 101, 1001–1008 10.1161/CIRCRESAHA.107.160812 17885215

[B30] Zhan Y. Paolicelli R. C. Sforazzini F. Weinhard L. Bolasco G. Pagani F. (2014). Deficient neuron-microglia signaling results in impaired functional brain connectivity and social behavior. Nat. Neurosci. 17, 400–406 10.1038/nn.3641 24487234

